# Laser surface structuring of diamond with ultrashort Bessel beams

**DOI:** 10.1038/s41598-018-32415-0

**Published:** 2018-09-19

**Authors:** Sanjeev Kumar, Shane M. Eaton, Monica Bollani, Belén Sotillo, Andrea Chiappini, Maurizio Ferrari, Roberta Ramponi, Paolo Di Trapani, Ottavia Jedrkiewicz

**Affiliations:** 1 Department of Science and High technology, Università dell’Insubria, via Valleggio 11, 22100 Como, Italy; 2 Institute for Photonics and Nanotechnologies, CNR and Department of Physics, Politecnico di Milano, Piazza Leonardo da Vinci 32, 20133 Milano, Italy; 3 Institute for Photonics and Nanotechnologies, CNR, L-NESS, Via Anzani 42, 22100 Como, Italy; 4 Institute for Photonics and Nanotechnologies, CNR, CSMFO Lab., Via alla Cascata 56/C, Povo, Trento 38123 Italy; 5 Institute for Photonics and Nanotechnologies, CNR, Udr Como, Via Valleggio 11, 22100 Como, Italy

## Abstract

We investigate the effect of ultrafast laser surface machining on a monocrystalline synthetic diamond sample by means of pulsed Bessel beams. We discuss the differences of the trench-like microstructures generated in various experimental conditions, by varying the beam cone angle, the energy and pulse duration, and we present a brief comparison of the results with those obtained with the same technique on a sapphire sample. In diamond, we obtain V-shaped trenches whose surface width varies with the cone angle, and which are featured by micrometer sized channels having depths in the range of 10–20 *μ*m. By laser writing crossed trenches we are also able to create and tailor on the diamond surface pillar-like or tip-like microstructures potentially interesting for large surface functionalization, cells capturing and biosensing.

## Introduction

Diamond is the hardest material known and thanks to its exceptional properties such as high thermal conductivity, wide bandgap, very good optical properties and biocompatibility, it is increasingly used for different applications such as photonics^1–3^, quantum information technologies^4–6^ and microfluidics and biosensing^6–8^.

Different fabrication methods, often lengthy and complex, have been used so far for diamond surface microstructuring, such as mold techniques with the use of sacrificial layers^9–11^, focusing ion beam technology^12,13^, or reactive ion etching^14^. Generally because of its hardness, efficient fabrication methods for diamond are still limited, especially for cases where sub-micron or micron size surface structures are needed. On the other hand, laser microfabrication has been mainly limited to the investigation of the periodical sub-micron ripples observed on the sample surface after laser irradiation^15–20^ and to the generation of channel-like structures with an average depth of less than half micron^21^. In this context we have recently shown the possibility to use the laser micromachining technology^22^ combined with a beam shaping technique to generate ultrashort pulse Bessel beams, for a deep surface ablation of diamond in a single pass^23^.

The Bessel beam (BB) featured by a central peak (Bessel core) surrounded by rings (conical energy reservoir), is a particularly interesting class of light beams, which can be generated by conical lenses (axicons) or holograms. Thanks to their non-diffracting properties they have been used in various research areas^24^, ranging from extreme nonlinear optics^25^, particle trapping^26^ to laser micromachining^27–36^, or fluorescence imaging^37^. The non-diffracting behavior of the Bessel beams results into a long depth of field, which can be used to increase the tolerance of the focal plane position during material processing, especially in situations where in-depth machining is required. Pulsed Bessel beams have already been used for bulk generation of graphitic microstructures in diamond^38^, while in ref.^23^, a 200 fs BB with a given cone angle was used in a transverse writing configuration in such a way to generate an ablation pattern on the diamond substrate surface, featured by 3D V-shaped surface trenches.

While the work reported in ref.^23^ presented the first results of the BB machining experiment obtained as a function of different writing speeds, for single pass tracks, the aim of this paper is to study the possibility of tailoring the geometry and features of the surface microchannels that can be generated on diamond as a function of the beam cone angle used, the pulse duration and the pulse energy. Moreover by optimizing the laser writing parameters and the writing configuration we will demonstrate the laser fabrication of more complex structures, such as pillar-like microstructures, never reported to date on diamond by means of this technique and finding applications in a wide range of fields, such as fluorescent imaging, biosensing^39^, or for cell growth adhesions^40^, cell isolation^41^ and DNA purification^42,43^. Note that in comparison to the micropillars created on diamond by means of lengthy techniques such as photolithography and plasma etching^44^ or focused ion beam^45^, the localized 3D microstructures obtained in this work by means of a fast BB laser writing process can be closely spaced and thus distributed with a high density and can be generated with different features and geometries. Finally the surface microstructured trenches that can be generated with the above mentioned technique are also compared with the results obtained on sapphire, a crystalline material with hardness and density close to diamond.

## Results

**Figure 1:**
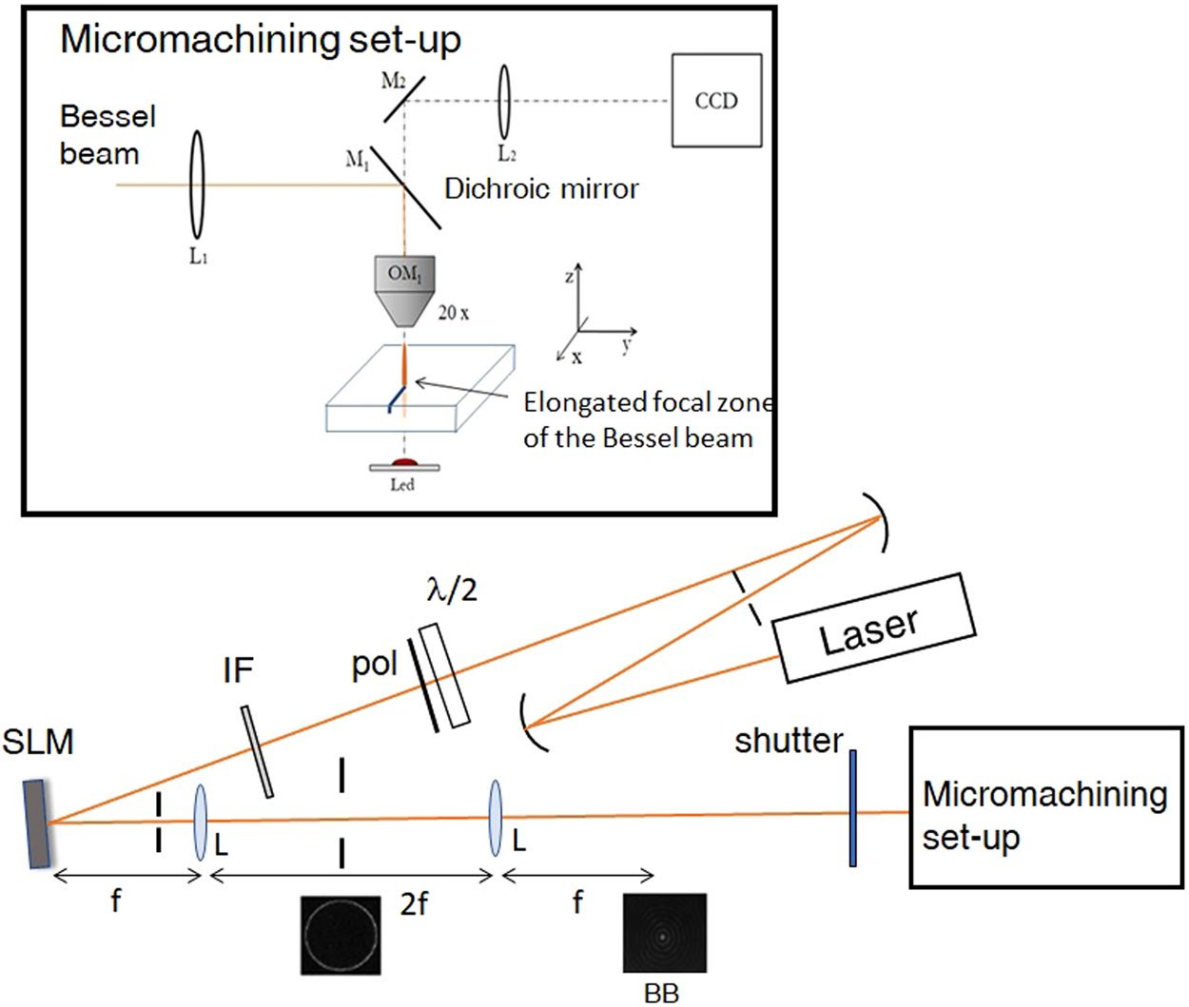
Experimental apparatus for the surface laser microfabrication of a synthetic diamond sample by means of a Bessel beam. In the inset, detailed micromachining set-up with the imaging system for real-time surface sample observation.

**Figure 2:**
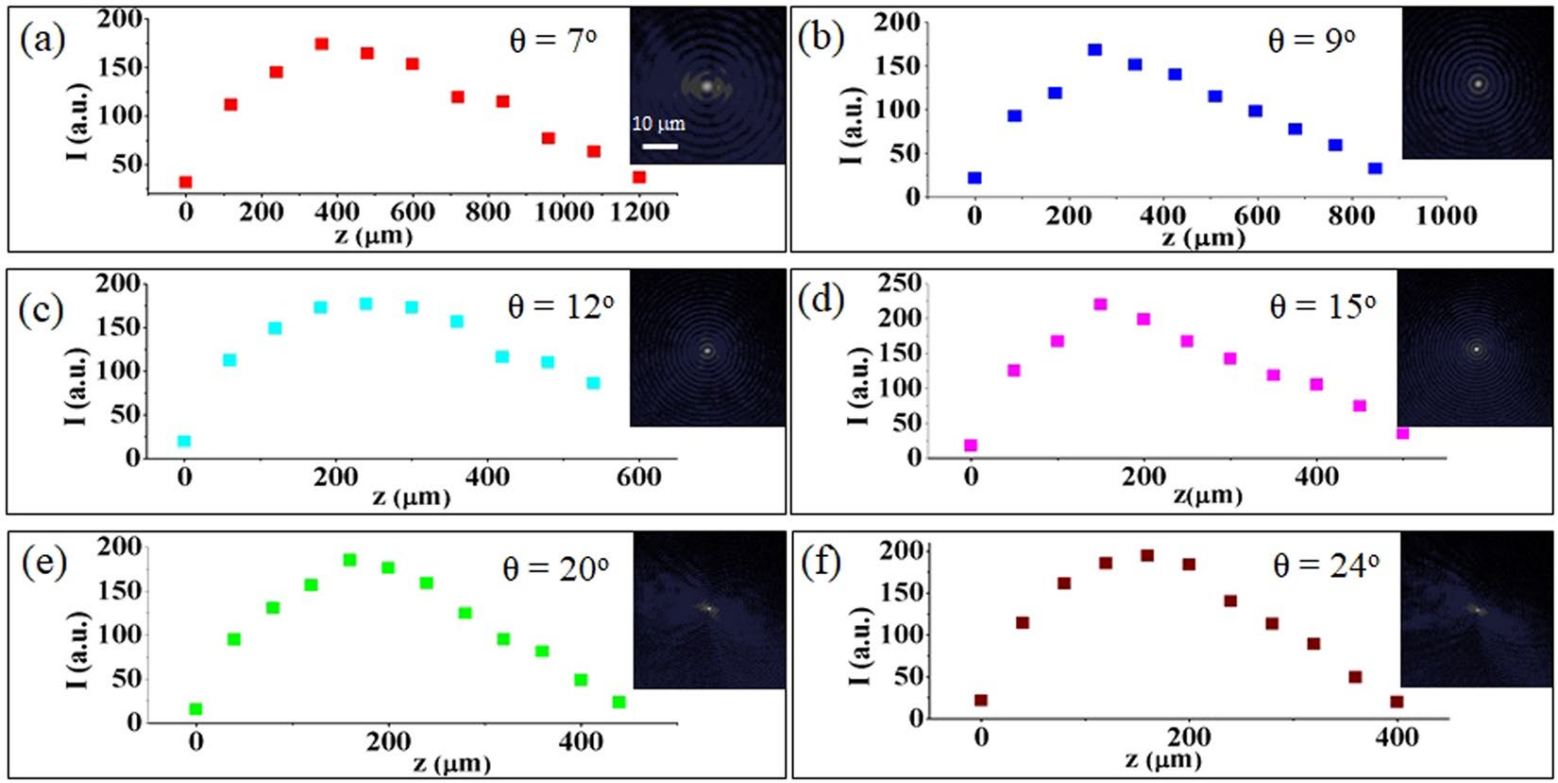
On-axis Bessel beam peak intensity evolution along the propagation direction for a beam cone angle *θ* = 7° (**a**), 9° (**b**), 12° (**c**), 15° (**d**), 20° (**e**) and 24° (**f**). In the insets, the corresponding BB patterns recorded by a CCD camera in the center of the Bessel zone. The scale bar corresponding to 10 *μ*m is the same for all images.

The microfabrication experiments have been performed with a pulsed laser (see Methods) and using a laser machining set-up where the BB, generated by a spatial light modulator (SLM) and then demagnified by a telescopic imaging system, is directed perpendicularly onto a 500 *μm* thick diamond sample, for transverse writing. The experimental apparatus is shown in Fig. 1. The microfabrication part of the set-up is featured by a motorized translation stage allowing the micrometer control of the sample movement via software^23^. The use of the SLM allows to change the BB cone angle *θ* in a versatile way and therefore also the non-diffractive zone length (Bessel zone) *z*_*max*_ = *w*_0_/*tanθ* and the Bessel core radius size *r*_0_ = 2.40408*λ*/2*πsinθ*, *w*_0_ being the transverse size of the input beam on the SLM evaluated as full width at half maximum (FWHM) from the intensity transverse profile. For illustration, the transverse spatial profile of the BB obtained with different cone angles and recorded by a CCD camera, together with the evolution of its peak intensity along the Bessel zone in air are presented in Fig. 2. Central core and Bessel zone are smaller for larger cone angles. Also note that because of the different refractive index of the material, a further elongation of the Bessel beam inside the sample bulk occurs. Thus thanks to the elongated focal zone of the BB there is no need to shift the sample along the beam direction for extended vertical machining in contrast to the requirement of Gaussian beam processing.

### Single-shot and multiple-shot Bessel beam machining on diamond surface

**Figure 3:**
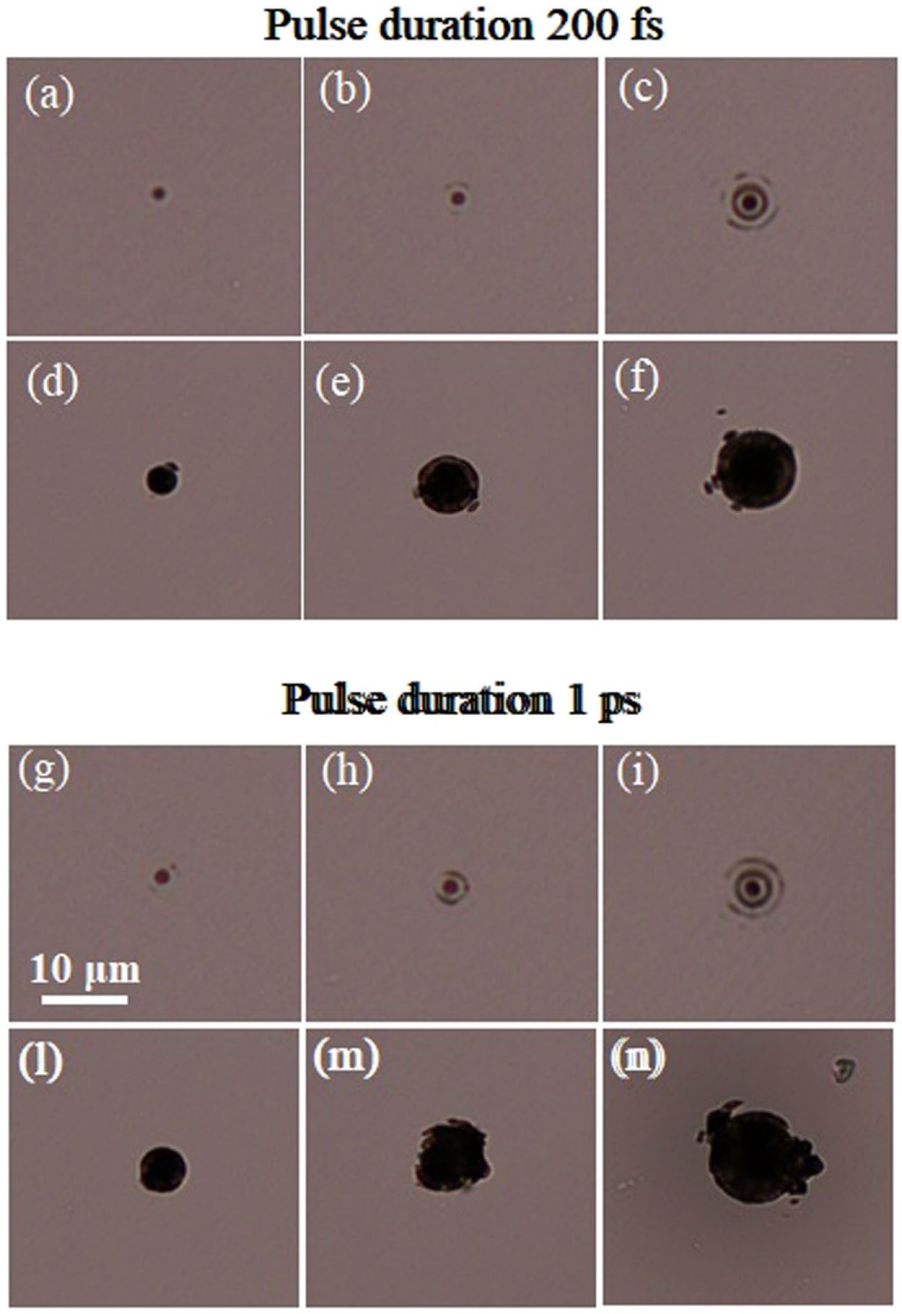
Top panel: Optical microscope images of single shot (**a**–**c**) and multiple shot (**d**–**f**) traces left by a 200 fs pulsed BB on the diamond surface for increasing pulse energy. Bottom panel: Optical microscope images of single shot (**g**–**i**) and multiple shot (**l**–**n**) traces left by a 1 ps pulsed BB on the diamond surface for different pulse energies. The multiple shot machining corresponds to 280 spatially superimposed pulses; the BB cone angle is 20°. Pulse energy used was *E* = 3.5, 7 and 10 *μ*J in the first, second and third column respectively. The 10 *μ*m scale bar indicated in (**g**) is the same for all images.

The very first experiment consisted in studying the ablation features on the diamond surface produced by the BB orthogonally impinging on the material. The sample was aligned in such a way to have the most intense portion of the elongated BB focus crossing its top surface. After the micromachining process, one could observe under the optical microscope different kinds of traces left by the high intensity BB core and in some cases by the surrounding rings. The pulse energy can be optimized to have, even in single shot, a single homogeneous disk-shape mark due to the nonlinear absorption of the central core only, or to have an ablation trace reflecting the Bessel beam transverse profile due to the nonlinear absorption of the core and a few external rings. In Fig. 3, we present optical microscope images of single shot traces and multiple shots traces generated on the top surface by respectively a 200 fs and 1 ps pulsed BB, with a cone angle of 20° and a central core of 0.7 *μ*m, evaluated as full width at half maximum. The images (a), (b) and (c) in the first panel and (g), (h), (i) in the second panel are relative to single shot ablation, while the others are multiple shot images obtained with 280 spatially superimposed pulses. From left to right the pulse energy is increasing and we calculated that in the energy range used, the fluences associated with the BB core are of the order of a few J/cm^2^ (from 2 to 6 J/cm^2^) thus above the diamond damage threshold^46^.

**Figure 4:**
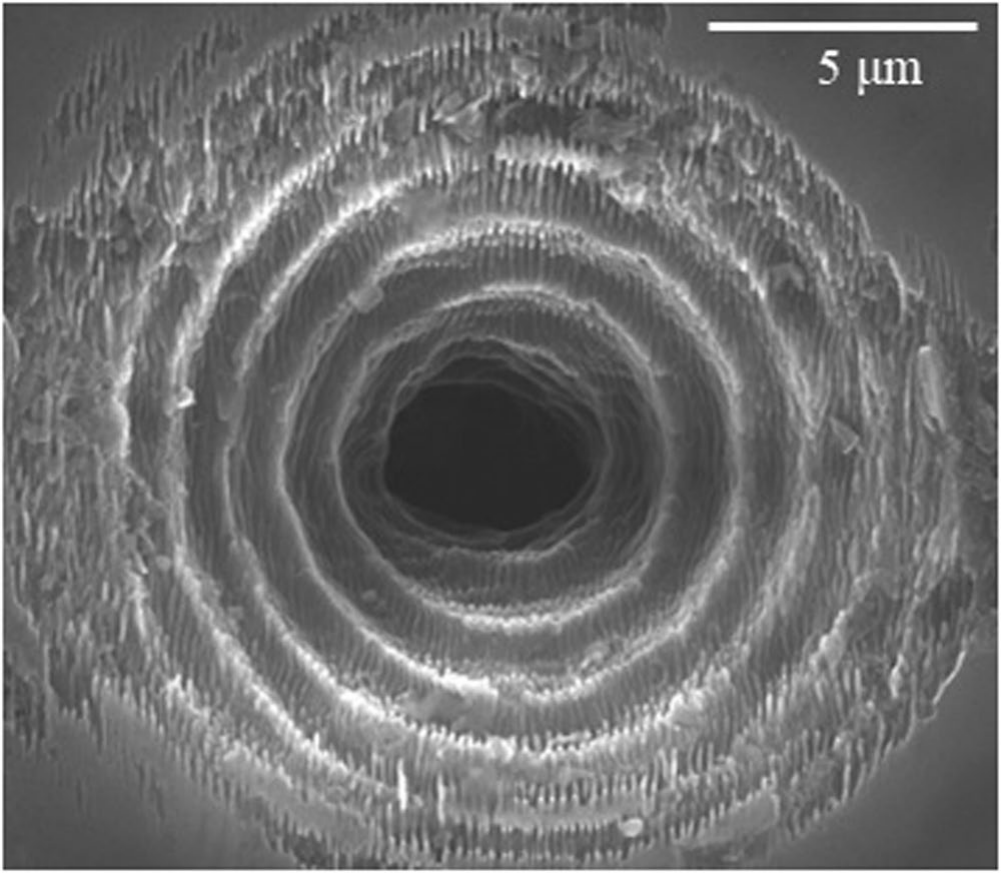
Planar view SEM image of a multiple shot surface trace obtained after laser irradiation of the diamond sample by 500 spatially superimposed Bessel pulses. The beam cone angle is 20° and the pulse energy 10 *μ*J.

While no substantial difference can be observed in single shot for the two different pulse duration cases, in multiple shot when using a picosecond pulse, additional thermal effects lead to a more inhomogeneous and stronger ablation pattern. The black traces left on the surface (see Fig. 3) especially in the multiple shot regime, are associated with the presence of a surface graphitic phase generated during the laser irradiation. The presence of this graphitic-like phase was confirmed by micro-Raman spectroscopy measurements as reported in^23^. A wet chemical etching with a solution of H_2_SO_4_ (96% vol): HNO_3_ (69% vol): H_2_O (with volume ratio 8:4:1) can therefore be performed to clean the sample and remove the possible graphitic contamination without attacking the diamond substrate^47^. By scanning electron microscopy (SEM), we are able to characterize more in detail the micro-ablation features generated after the BB laser irradiation. In Fig. 4 we report for illustration a SEM image (taken after chemical cleaning) of a multiple shot trace left at the top surface of the diamond sample by a 200 fs pulsed BB with 20° cone angle and 10 *μ*J pulse energy. While the central portion of the damaged material is featured by a hole ablated by the central core of the BB, one can also observe the traces left by a few lateral rings. On the other hand we notice the presence of nanogrooves, as observed before always orthogonal to the laser polarization^17,23^, which are more evident on the regions of ablated material deriving from lower intensity portions of the impinging beam.

### Surface microchannels by single pass Bessel beam laser writing

To extend the work in^23^ we have studied the effect of the BB geometry on the features of the channel-like microstructures that can be generated in single pass by laser writing. Here we shall concentrate on the femtosecond pulsed regime which enables the most precise microfabrication by avoiding heating and extended shock wave effects during the radiation-matter interaction process^22^, but we will also briefly comment on results obtained in the picosecond regime. During the laser irradiation, the sample was shifted in the horizontal plane (parallel to its surface) orthogonally to the BB (impinging from the top) in order to write trenches in a single pass. The speed of translation was optimized in such a way to homogeneously ablate the sample surface in depth^23^ and corresponded to having 280 spatially superimposed pulses per position.

#### Femtosecond regime

**Figure 5:**
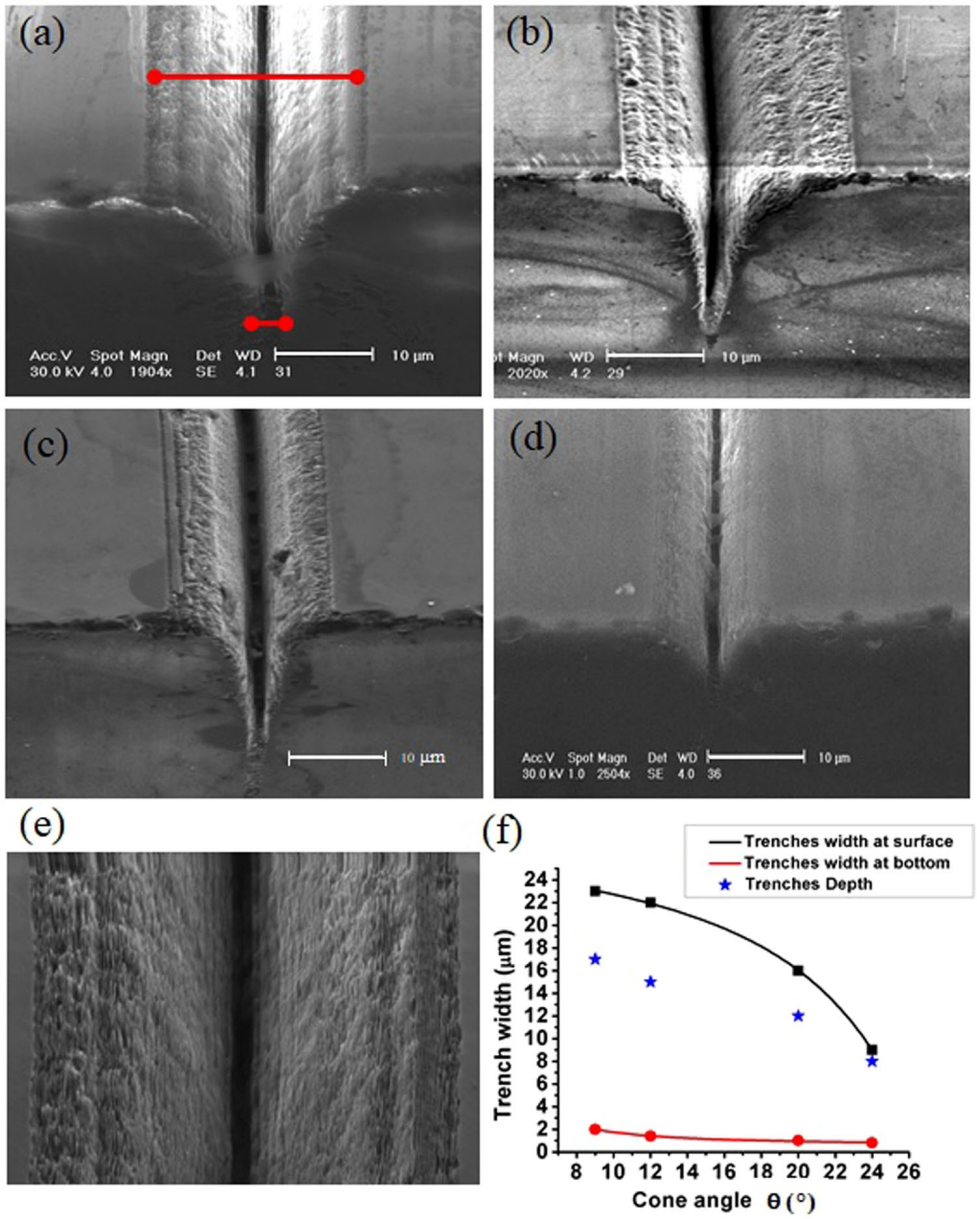
Tilted view SEM images of microtracks machined in a single pass on the diamond surface by means of a 200 fs pulsed BB, with different cone angles and energies (**a**) *θ* = 9°, *E* = 5.4 *μ*J, (**b**) *θ* = 12°, *E* = 4.5 *μ*J, (**c**) *θ* = 20°, *E* = 3.5 *μ*J and (**d**) *θ* = 24°, *E* = 3 *μ*J. The scale bar for all SEM images is 10 *μ*m. In all cases the writing speed corresponded to having 280 spatially superimposed pulses. In (**e**), top view SEM image of a zoomed portion of (**a**). In (**f**), evolution of the geometrical features of the V-shaped track as function of the cone angle. The continuous curves are fits of the collected data. The scale bar in the images (**a**–**c**) and (**d**) corresponds to 10 *μ*m, while in (**e**) the scale bar corresponds to 5 *μ*m.

Figure 5 presents SEM images of 80 *μ*m long trenches written with BBs featured by different cone angles and shows the effect of these on the V shape of the obtained trenches. The pulse duration was 200 fs and the fluence was held fixed at 2 *J*/*cm*^2^. This means we slightly reduced the energy for increasing BB cone angle and decreasing core size. A rough analysis of the V-shape features of the trenches generated in different conditions has been carried out from the SEM images together with an evaluation of the trenches depth and their width at the surface and at the bottom of the ablated tracks (Fig. 5(f)), simply to highlight the differences appearing for different cone angles. The red marks in Fig. 5(a) indicate how these features have been extracted from the SEM images. As the BB cone angle increases, the rings surrounding the central core get closer (see Fig. 2). Consequently for fluences above the ablation threshold, the surface ablation traces are globally featured by smaller transverse extensions for larger BB cone angles. One can also observe that in this case the walls of the V-shaped trenches are more vertical than in the case of small cone angles, while their depth slightly decreases. Note that in general the presence of an extended collateral damage around the tracks due to the beam side lobes is useful for the generation of differently shaped pillar-like microstructures in three dimensions (3D), implemented by crossing the writing trajectories. This will be shown in the last section before the discussion. Figure 5(e) is a top view SEM image of a zoomed portion of Fig. 5(a), showing the microchannel generated in the center of the ablated track and the presence of nanogrooves already highlighted in ref.^23^, always orthogonal to the laser polarization and thus with a well controllable orientation.

#### Picosecond regime

**Figure 6:**
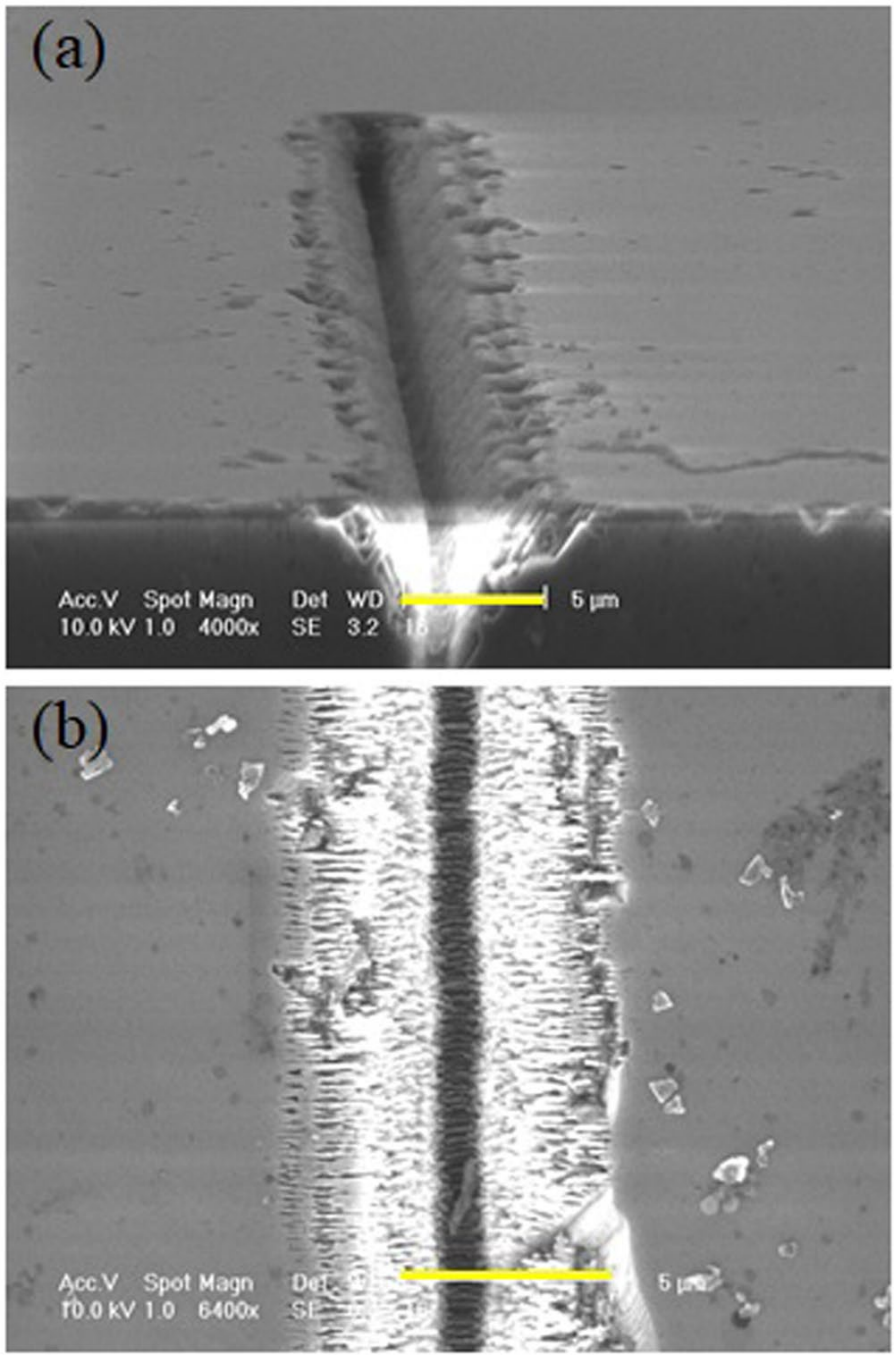
SEM images: Tilted view (**a**) and zoomed top view (**b**) of a surface microtrack generated on diamond by means of a 1 ps pulsed BB with cone angle *θ* = 20° and laser pulse energy *E* = 3.5 *μ*J. The writing speed corresponded to having 280 spatially superimposed pulses per position. The scale bars in (**a**) and (**b**) here corresponds to 5 *μ*m.

We repeated the experiment in the picosecond regime and for a direct comparison with the previous results we used similar fluences and the same writing speed. The results show a degradation of the quality and precision of the trenches generated on the diamond surface. Figure 6 shows as an example a tilted view and a top view of a microtrack generated with a 3.5 *μ*J, 1 ps pulsed BB with cone angle *θ* = 20°. We also notice that the depth of the microstructure appears to be smaller than that of the one presented in Fig. 5(c) for the same fluence. This may be attributed to the lower local peak intensity of the impinging beam. On the other hand we notice in Fig. 6 that the walls of the internal microchannel are featured by a less pronounced V shape, but are rather vertical, probably due to a reduced contribution to the ablation from the rings. Irregular structures are present at the lateral edges of the ablated regions and signs of microexplosions with presence of debris are evident. We attribute this to the stronger thermal effects involved when the avalanche ionization, which occurs on the picosecond time scale, dominates during the nonlinear absorption process^22^.

### Comparison with sapphire

The Bessel beam ablation technique was also tested on another biocompatible material, sapphire, the scope being a qualitative comparison of the response of this material when irradiated by an elongated focused beam across the top surface. By laser machining transparent samples with different physical properties, such as melting temperature, thermal conductivity, thermal expansion coefficient, hardness and density, one expects to obtain different features for the generated surface microstructures.

**Figure 7:**
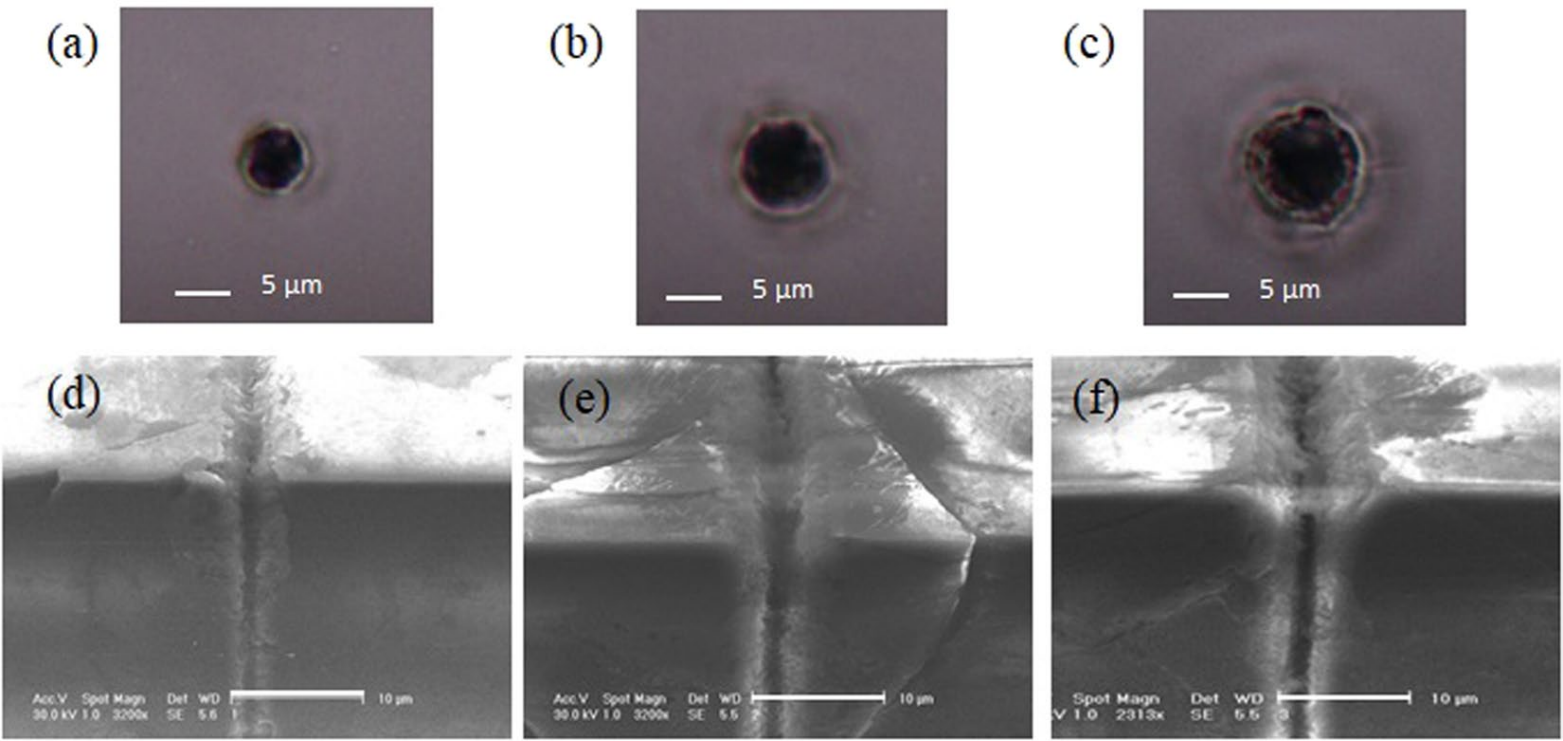
Top row: Optical microscope images of multiple shot traces (280 superimposed pulses) left on a sapphire sample by a 15° Bessel beam, with pulse energy of 10 *μ*J (**a**), 15 *μ*J (**b**) and 20 *μ*J (**c**); Bottom row: Tilted view SEM images of surface microstracks written by translating the sample with a speed corresponding to having 280 superimposed pulses per position and a BB pulse energy of 10 *μ*J (**d**), 15 *μ*J (**e**) and 20 *μ*J (**f**) respectively. The scale bars in (**d**,**e**) and (**f**) correspond to 10 *μ*m.

Figure 7 shows in the top row, optical microscope images of multiple shot traces left on a 450 *μ*m thick monocrystalline c-plane sapphire sample by a 200 fs, 15° Bessel beam with different pulse energies and in the bottom row SEM images of surface microtracks obtained in the same corresponding energy conditions. The traces in Fig. 7(a–c) present a similar disk-like shape as those on diamond, but here small lateral cracks can be observed especially for higher energies. These cracks are evident at the sides of the corresponding top surface trenches shown in Fig. 7(d–f). Note that because the refractive index of sapphire (1.7) is smaller than that of diamond (2.4), a smaller BB cone angle in air was used in order to have an internal refractive angle (of the lateral incoming rays belonging to the rings) similar to the case of diamond and also to work with a similar Bessel zone length inside the bulk. Also the choice of a higher pulse energy derived from the fact that the ablation threshold fluence in sapphire (in the range of 6–8 J/cm^2^) is higher than in diamond^48,49^ and was optimized to produce the best possible quality channel-like structures. The result was the generation of deeper trenches with respect to diamond, which can be partially attributed to the formation of a graphitic phase having an absorption coefficient substantially larger than in different forms of mono- and polycrystalline diamond, which reduces the optical transmission of the beam^50^. Moreover we may also partially attribute the deeper trenches in sapphire to a slightly different positioning of the BB focal length with respect to the sample, as the experiment was performed at a different time than that on diamond. Nevertheless in comparison to diamond, the internal features of these structures turned out always to be much less homogeneous. Although the hardness and density of sapphire are comparable to those of diamond, its lower melting temperature and its higher thermal expansion coupled to a much lower thermal conductivity^51^ may be responsible both for the inhomogeneously localized material redeposition and for the observed cracks departing from the laser machined structures. Indeed the cracking phenomenon in sapphire is difficult to avoid as it is due to tensile stress generated within the material by the high energy density deposited, in agreement with previous observations^52^.

### Bessel beam laser writing of pillar-like microstructures

The possibility to write tailored microtrenches and microchannels with nanometer-size substructures on a biocompatible material, such as diamond, is an important result for the realization of ad hoc lab-on-chips useful for microfluidic or biosensing applications^53^. Importantly, diamond can be very easily and reliably functionalized with organic molecules, DNA or enzymes and it is suitable for the attachment of circulating tumor cells (CTC)^54,55^. The possibility to increase the area to be functionalized is often performed by fabricating micropillars on the material surface^44,45,56^. While these are typically obtained by chemical etching lithography or ion beam lithography and thus by means of lengthy processes, we have investigated a way to generate similar microstructures by BB laser writing, never reported so far in diamond. Note that micropillars packing density and top surface area can influence cellular adhesion and its response^57^. Therefore, the diameter of individual micropillars and spacing between them are two crucial parameters. Here, to create closely spaced 3D microstructures with tailorable features and different shapes (pillar-like or tip-like), we performed a laser machining of the diamond surface in transverse configuration by orthogonally crossing the writing trajectories and with varied laser writing parameters, with the advantage of a faster processing with respect to more standard lithographic techniques.

**Figure 8:**
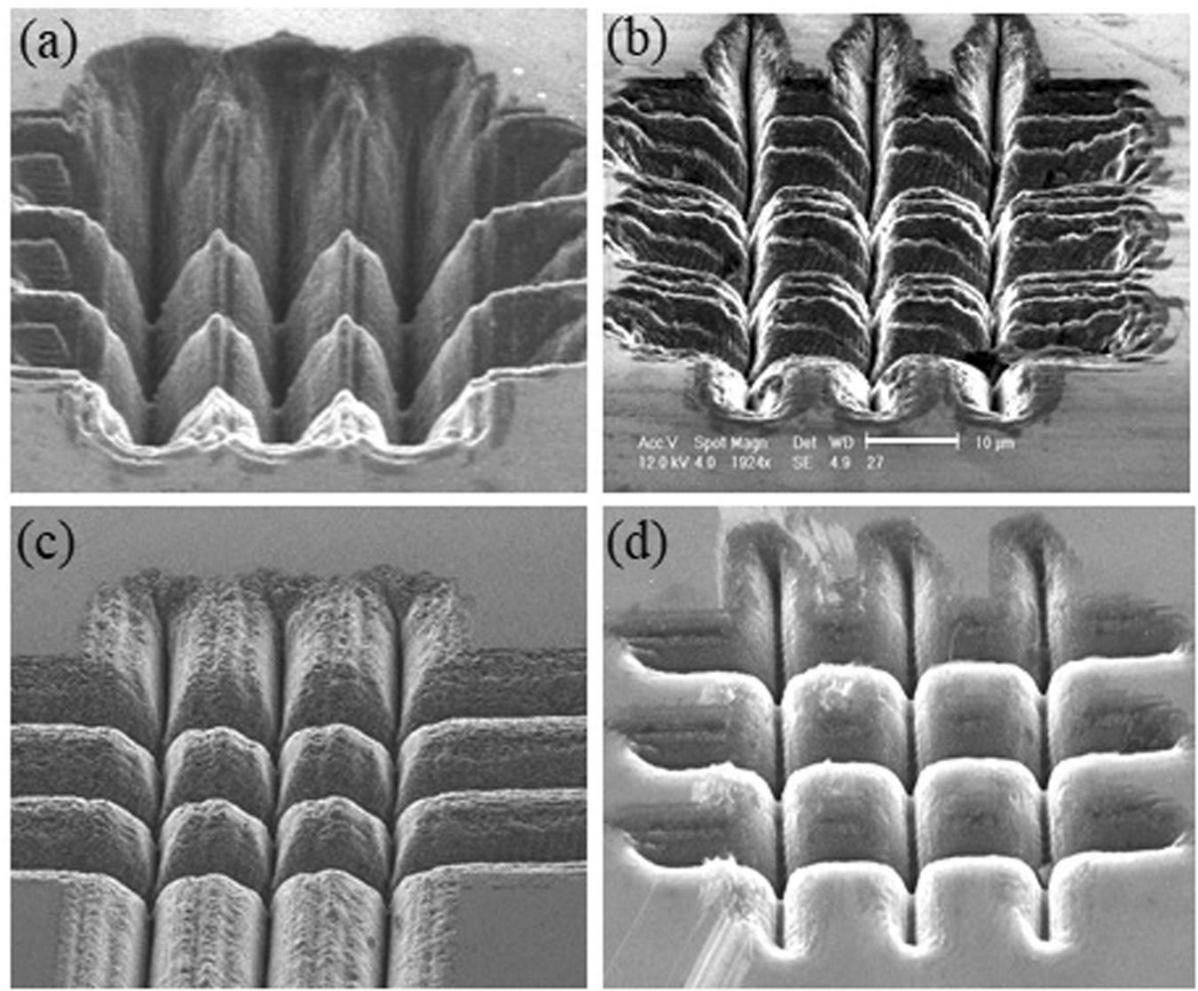
SEM images of pillar-like microstructures arrays written on a diamond surface by a 200 fs pulsed Bessel beam in transverse writing configuration and for different cone angles and pulse energies (**a**) *θ* = 9°, *E* = 5.4 *μ*J, (**b**) *θ* = 12°, *E* = 4.5 *μ*J, (**c**) *θ* = 20°, *E* = 3.5 *μ*J and (**d**) *θ* = 24°, *E* = 3 *μ*J. Distance between the writing trajectories is 15 *μ*m. The 10 *μ*m scale bar shown in (**b**) is the same for all images.

**Figure 9:**
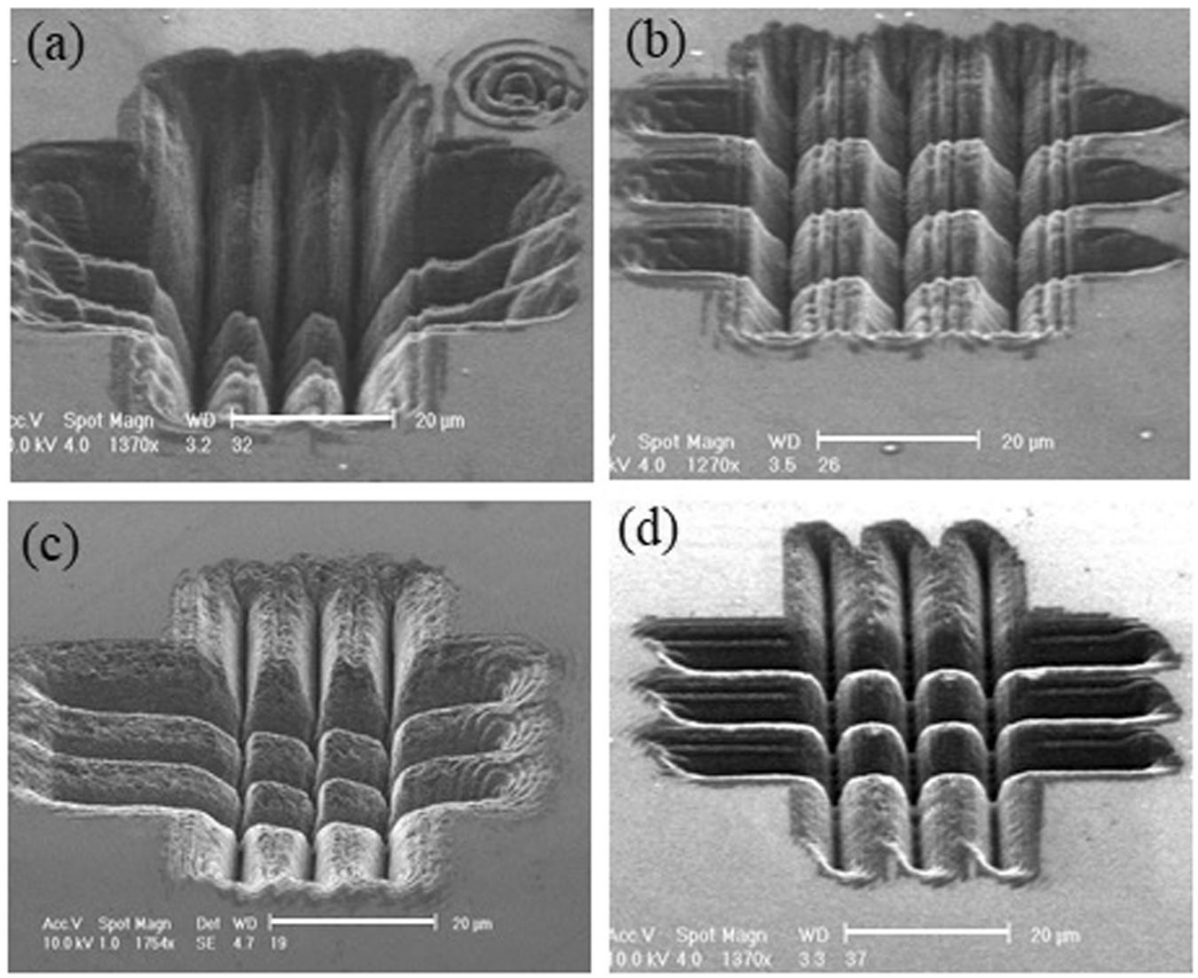
SEM images of pillar-like microstructures arrays written on a diamond surface by a 200 fs pulsed Bessel beam in transverse writing configuration and for different cone angles and pulse energies (**a**) *θ* = 9°, *E* = 5.4 *μ*J, (**b**) *θ* = 12°, *E* = 4.5 *μ*J, (**c**) *θ* = 20°, *E* = 3.5 *μ*J and (**d**) *θ* = 24°, *E* = 3 *μ*J. Distance between the writing trajectories is 10 *μ*m. The scale bars in all images here correspond to 20 *μ*m.

The SEM images presented in Figs 8 and 9 show the microstructures obtained in the femtosecond regime in a three-by-three crossed single pass trench-writing configuration, for different BB cone angles (and accordingly slightly different pulse energies to keep the pulse fluence approximately constant) and for two different pitch values (separation distance between two writing trajectories), namely 15 *μ*m (Fig. 8(a–d)) and 10 *μ*m (Fig. 9(a–d)). Differently shaped localized microstructures appear for different irradiation regime and BB features. We observe in Fig. 8(a–d) that for a given pitch, the spacing between the edges of the pillar-like microstructures decreases as the cone angle of the beam increases. On the other hand the pillar’s average width increases with the cone angle and the microstructure’s tip becomes less sharp. The morphology of these microstructures strongly depend on that of the ablated surface trenches. For larger values of the cone angle, one can fabricate pillars which present a smoother and more regular surface, with more vertical walls. We also observe that by increasing the beam cone angle, it is possible to obtain more tip-like microstructures with a modulated surface pattern as in Fig. 8(a,b), pillar-like microstructures where nanofeatures entirely characterize the surfaces as in Fig. 8(c) and finally cubic microstructures with a flat surface (Fig. 8(d)). Note that a similar trend is observable in the images of Fig. 9 described just below. Clearly the nanogrooves appearing on the trenches and thus on the pillar walls will affect their surface properties. The possibility to control the roughness and the micro and nano features of the pillars is an added value for all those biosensing applications, where cell adhesion will depend on the sharpness and particular roughness or smoothness of the pillar tip. The dimension of the pillars depends also on the pitch value chosen during the laser writing process and therefore on the gap between the generated trenches. In Fig. 9 where the writing pitch has been set equal to 10 *μ*m and the pillar-like structures are smaller, we notice in one case (Fig. 9a) a strong collapse of the whole machined structure as the resulting ablated trenches are strongly overlapping. The effect is reduced for larger cone angles and disappears for the largest cone angle used (see Fig. 9d), where a regular micropillars array appears as consequence of the possibility to fabricate deeply ablated vertical microtrenches. In general the dimensions of our resulting localized 3D microstructures, which can be featured by a depth of at least 10 *μ*m are deeper than those created on diamond by more standard lithographic^44^ or focused ion beam^45^ techniques only a few *μ*m. Note that here the pillar-like microstructures generated by this laser writing technique are naturally distributed with a high packing density.

## Discussion

The results presented in this work show the possibility to fabricate various channel-like and pillar-like microstructures on the diamond surface by laser micromachining, an alternative fabrication technique that is faster than standard lithographic methods. The microfabrication technique presented here is based on a single pass transverse writing process making use of pulsed Bessel beams, whose geometrical features can easily be tuned by means of a spatial light modulator. The elongated focal zone of the BB allows to deeply ablate transparent materials without moving the sample along the beam direction, guaranteeing a faster machining process with respect to standard Gaussian beam laser processing. The effect of different beam cone angles, different pulse energies and also femtosecond and picosecond pulse durations has been investigated, with the results highlighting the possibility to easily tailor the surface features of the ablated structures on the micro-nano scale and to obtain channel-like microstructures useful for microfluidics applications. A comparison with the results obtained in Sapphire has highlighted the high level of writing precision obtainable in diamond. Once established the writing conditions for obtaining at least 10 *μm* deep microtracks on the diamond surface, as shown in this work, one can also fabricate in the same machining conditions pillar-like microstructures by simply crossing the laser writing trajectories. Here we have shown that the quality and features of the resulting micropillars arrays can be tailored again by tuning the geometrical features of the writing Bessel beam and the 3D localized structures that can be created are naturally distributed with a high packing density. Our results validate the use of this laser machining technique for an ad-hoc microstructuring process of biocompatible diamond surfaces in all cases where well-defined microstructures are needed, for instance for microfluidics or biosensing and cell capturing applications and thus also for all cases where an increase of the surface area to be functionalized is required.

## Methods

The experiment was performed by using a regeneratively amplified mode-locked Ti:Sapphire laser delivering 800 *nm*, 40 *fs* transform-limited pulses, at 20 Hz repetition rate. The pulse duration could be stretched by simply detuning the compressor system of the amplified laser system. The Gaussian laser beam, spatially filtered and demagnified was sent to a spatial light modulator (Holoeye) imprinting on the beam the phase mask of a conical lens. Note that a 10 nm bandwidth interference filter (IF) was placed before the SLM to reduce the pulse bandwidth and avoid beam distortions as the available SLM was not optimized for ultrashort pulses. The beam reshaped into a Bessel beam (BB) was then demagnified and imaged at the sample position, as described in^23^. In the micromachining part of the experimental set-up (see illustration in inset of Fig. 1) the BB was injected from the top, ortogonally to the (001)-oriented surface of a polished synthetic monocrystalline diamond sample (type *II*, 7 × 7 × 0.5 *mm*^3^, MB Optics). The diamond sample was then replaced by the sapphire sample for a comparison of the microtracks generated. Note that for the optimization of the surface microstructures obtained, the relative positioning of the BB with respect to the sample thickness was carefully adjusted taking into account that the intensity profile of the finite energy BB is not flat along the propagation direction (See Fig. 2).

All scanning electron microscopy (SEM) images have been carried out using a Philips XL30 SFEG SEM model working at 10 kV without any metal deposition on the sample surface, explaining in some acquisitions the presence of charging effects difficult to eliminate (see for instance Fig. 6).

## Data Availability

All data generated or analysed during this study are included in this published article.
